# Audit of a new model of birth care for women with low risk pregnancies in South Africa: the primary care onsite midwife-led birth unit (OMBU)

**DOI:** 10.1186/s12884-014-0417-8

**Published:** 2014-12-20

**Authors:** George Justus Hofmeyr, Thozeka Mancotywa, Nomvula Silwana-Kwadjo, Batembu Mgudlwa, Theresa A Lawrie, Ahmet Metin Gülmezoglu

**Affiliations:** Effective Care Research Unit, University of Fort Hare, Private Bag X9047, East London, South Africa; Walter Sisulu University, Mthatha, Eastern Cape South Africa; Eastern Cape Department of Health, Frere Maternity Hospital, Private Bag X9047, East London, 5201 South Africa; UNDP/UNFPA/UNICEF/WHO/World Bank Special Programme of Research, Development and Research Training in Human Reproduction (HRP), Department of Reproductive Health and Research, World Health Organization, Avenue Appia 20, 1201 Geneva, Switzerland

**Keywords:** Midwife-led unit, Primary birth care, Onsite birth unit, OMBU

## Abstract

**Background:**

South Africa’s health system is based on the primary care model in which low-risk maternity care is provided at community health centres and clinics, and ‘high-risk’ care is provided at secondary/tertiary hospitals. This model has the disadvantage of delays in the management of unexpected intrapartum complications in otherwise low-risk pregnancies, therefore, there is a need to re-evaluate the models of birth care in South Africa. To date, two primary care onsite midwife-led birth units (OMBUs) have been established in the Eastern Cape. OMBUs are similar to alongside midwifery units but have been adapted to the South African health system in that they are staffed, administered and funded by the primary care service. They allow women considered to be at ‘low risk’ to choose between birth in a community health centre and birth in the OMBU.

**Methods:**

The purpose of this audit was to evaluate the impact of establishing an OMBU at Frere Maternity Hospital in East London, South Africa, on maternity services. We conducted an audit of routinely collected data from Frere Maternity Hospital over two 12 month periods, before and after the OMBU opened. Retrospectively retrieved data included the number of births, maternal and perinatal deaths, and mode of delivery.

**Results:**

After the OMBU opened at Frere Maternity Hospital, the total number of births on the hospital premises increased by 16%. The total number of births in the hospital obstetric unit (OU) dropped by 9.3%, with 1611 births out of 7375 (22%) occurring in the new OMBU. The number of maternal and perinatal deaths was lower in the post-OMBU period compared with the pre-OMBU period. These improvements cannot be assumed to be the result of the intervention as observational studies are prone to bias.

**Conclusions:**

The mortality data should be interpreted with caution as other factors such as change in risk profile may have contributed to the death reductions. There are many additional advantages for women, hospital staff and primary care staff with this model, which may also be more cost-effective than the standard (freestanding) primary care model.

## Background

In developing countries, perceived poor quality of care and the medicalization of childbirth are both important barriers to facility-based birth [1]. A recent community-based survey of 1898 women living in rural, low income settings in the United Republic of Tanzania reported that 42% had bypassed their local primary care facility without a referral to give birth at a higher level of care, usually a government hospital [2]. Despite logistical challenges and added costs, bypassers were more likely than non-bypassers to report being very satisfied with the overall birth experience, and to rate the quality of care higher. It was concluded that gradually shifting birth care from primary care clinics to health centres and hospitals in this setting might improve health and experiential outcomes, as well as improving health system efficacy [2]. However, such a shift could overload the higher level of care services and result in unnecessarily medicalized and expensive care for healthy, low-risk, pregnant women. Against a backdrop of persistently high maternal mortality ratios in Sub-Saharan Africa, there is an urgent need to re-evaluate the models of birth care available to women in this region.

In South Africa, a middle income country, the national health system is based on the primary care model. The objective of this model is that the majority of people with less serious conditions receive care in a primary care setting. For women with ‘low-risk’ pregnancies, primary care clinics provide antenatal and postnatal care, and community health centres provide both antenatal/postnatal care and a 24-hour birth care service; women with ‘high-risk’ pregnancies receive doctor-led antenatal and birth care at secondary or tertiary care hospitals [3,4]. This model works well for antenatal care; however, the theory that women can be antenatally triaged to receive low-risk birth care at a primary care community health centre has two fundamental flaws in practice:

1. Intrapartum complications commonly arise unexpectedly in apparently low-risk women. Our experience in South Africa is similar to that reported in India, where about 30% of apparently low-risk women require referral to hospital during labour [5]. Referral rates have been reported to be over 20% in the United Kingdom (UK) [6,7], 15% in Denmark [8], 7-29% in Australia [9], 29% in Norway [10], and 42% in Japan [11].
2. Intrapartum complications are frequently very urgent problems requiring immediate intervention, e.g. cord prolapse, placental abruption, fetal distress, undiagnosed breech or twin pregnancy, shoulder dystocia, and postpartum haemorrhage. Even if the community health centre is only a few kilometers from the referral hospital, referral involves, at best, a very uncomfortable ambulance transfer for a woman in labour and, at worst, loss of life due to the seriousness of the condition or transport delays.

One of the consequences of this dilemma is that women who prefer to deliver in what may be perceived to be a ‘higher quality’ facility may use various strategies, such as arriving at the hospital in advanced labour in the hope that it will be too late to be sent away to deliver at the community health centre. Such strategies may result in birth before arrival at the hospital and put both mothers and babies unnecessarily at risk of adverse pregnancy outcomes.

Inevitably, there are some low-risk women who receive birth care in secondary and tertiary hospitals in South Africa. Therefore, in some of the larger hospitals, the obstetric unit (OU) is divided into ‘low-risk’ (midwives’) clients and ‘high-risk’ (doctors’) patients (some of whom have normal births). However, this model undermines the principle of primary care for low-risk pregnancies. In addition, low-risk women who are admitted to the OU are cared for by hospital staff and use expensive hospital resources. A study from New Zealand found that low-risk women receiving midwife care in a secondary care setting compared with a primary care setting had higher rates of caesarean section and admission of their babies to the neonatal intensive care unit [12]. Thus, once in an OU, there is an increased tendency for obstetric interventions to be utilized which are not appropriate for low-risk women, leading to a cascade of unnecessary interventions [13].

To enhance the normality of childbirth, many industrialized countries have introduced midwifery units which provide holistic, high quality, midwife-led care. This type of midwife-led care compared with doctor-led or shared care models has been found in a Cochrane review of 13 randomised controlled trials conducted in the UK, Australia, Canada, Ireland and New Zealand to be associated with an increased likelihood of a spontaneous vaginal birth, fewer obstetric interventions (instrumental delivery, episiotomy and epidural analgesia), and a trend towards greater maternal satisfaction and cost-effectiveness [14]. In the UK, depending on the location, women can choose to access midwife-led care in the form of ‘alongside midwifery units’ (AMUs) which provide midwife-led care based at the same site or in the same hospital as doctor-led obstetric units (OUs) [7]. Midwife-led care may also be provided on sites geographically separated from hospital OUs; these are known as ‘free-standing midwifery units’ (FMUs) [7]. Whilst the primary care birth service in South Africa may be staffed by midwives and provided in ‘free-standing’ centres, it is qualitatively very different from the UK model of midwife-led units in that it lacks the emphasis on ‘normality, continuity, advocating autonomy and building relationships with mothers’ [15] that is key to midwife-led models.

A commentary on the organisation of maternity care globally highlights the importance of sharing good practice models among countries and calls for research on effective ways of ‘revitalizing normal birth’, particularly in middle income countries [15]. In this paper, we propose a model of birth care adapted to the South Africa health system called the primary care onsite midwife-led birth unit (OMBU). Similar to AMUs in some respects (location and approach), it combines the benefits of giving birth on the premises of a hospital, with those of a midwife-led, low-cost, and low-intervention primary care birth. A unique aspect of the OMBU is that it is staffed, administered and funded by the primary care services, not the hospital. This makes it a useful model for countries where primary care is distinct from secondary/tertiary services, as is the case in many low-income countries such as Tanzania [2].

### The main features of the OMBU are as follows:

- ▪ Midwife-led unit for primary birth care similar to that typically provided in a 24-hour community health centre.
- ▪ Staffed, administered and funded by the primary care services.
- ▪ Provides birth care only, not antenatal care.
- ▪ Well suited to metropolitan areas with high population density, where women can get to a hospital as easily as to a community health centre.
- ▪ Located within a hospital, in close proximity to the obstetric unit/labour ward.
- ▪ Care provided follows the primary care model, including short-stay birth care with discharge six hours after birth if all is well.
- ▪ Less clinical approach is encouraged, with mobility in first stage, promoting birth companions and offering a choice of birthing postures.
- ▪ Obstetric interventions which might be employed by midwives include the artificial rupture of membranes, episiotomy, intramuscular opioid injections, and electronic fetal monitoring if indicated.

As a potential solution to excessive overcrowding in the OU, the first OMBU in South Africa’s Eastern Cape was established at Dora Nginza Hospital on the recommendation of two of the authors (GJH and N S-K). Subsequently, another OMBU was established at Frere Hospital in East London, South Africa, in March 2012. Frere Hospital is a referral hospital for a large drainage area of the Eastern Cape. With the OMBU model, low-risk women still receive antenatal care at the primary care clinics and community health centres but have the choice to give birth at the community health centre or at the hospital OMBU.

Prior to the establishment of the OMBU, a patient survey was carried out by one of the authors to assess women’s’ attitudes to an OMBU at the East London Hospital Complex (B Mgudlwa, unpublished Fellow of the College of Obstetricians and Gynaecologists research report). Most women surveyed were willing and able to access such a unit, with the associated added transport costs not considered a significant barrier to access. The new OMBU at Frere Hospital is currently staffed by birth care teams consisting of an operational manager, four midwives, a nurse and a nursing assistant. The unit includes five delivery beds, six postnatal beds and one newborn resuscitation station. The objective of this audit was to evaluate the impact of the establishment of the OMBU at Frere Maternity Hospital on maternity services.

## Methods

This audit used routinely collected data submitted to the Provincial Health Service on a monthly basis. The data were collected in the same way by dedicated staff of the OMBU and hospital labour ward and analysed retrospectively by the authors. The reliability of data sets before and after the intervention were thus comparable. Total number of births, maternal and perinatal mortality data, as well as mode of delivery data for the audit were collected retrospectively from Frere Hospital for the 12 month period from 1 January 2011 to 31 December 2011 before the OMBU opened, and for the 12 month period 1 July 2012 to 30 June 2013 after the OMBU opened. We allowed three months on either side of the March 2012 OMBU opening to ensure that the audit periods were a good representation of the situation.

## Results

**Figure 1:**
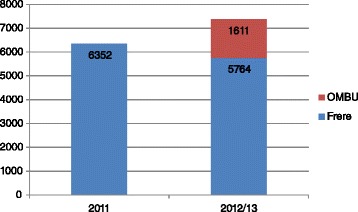
**No. of births at Frere Maternity Hospital 12 months before (2011) and 12 months after (2012/13) the opening of the OMBU.**

The total number of births on the hospital premises increased from 6352 during the earlier 12 month period to 7375 after the OMBU was established, representing a 16% increase. The number of births in the hospital OU decreased from 6352 to 5764 births, representing a 9.3% reduction, with 1611 women giving birth in the OMBU (22% of total births, excluding women referred to the OU due to complications arising during labour). Despite the increase in the total number of onsite births, the number of maternal deaths decreased from 14 deaths during the earlier 12 month period, to six deaths following establishment of the OMBU, equivalent to maternal mortality ratios of 220 and 81/100000 pre-and post-OMBU, respectively. Similarly, there was a reduction in perinatal mortality rates from 43 to 34 per 1000 births pre- and post OMBU (see Table 1 and Figure 1). Caesarean section rates were lower after the OMBU was established, at 35% of deliveries compared with 38%. These results should be interpreted with caution as various factors may have contributed to the observed reductions. These include changes in the risk profile and improvements in services unrelated to the OMBU.

**Table 1:** **Results of the Frere Maternity Hospital audits before and after opening the OMBU**

	**Frere OU 2011**	**Frere OU 2012/3**	**OMBU 2012/3**	**Frere OU + OMBU 2012/3**
Mothers giving birth	6352	5764	1611	7375
Babies born	6470	5875	1613	7488
Perinatal deaths	268	250	2	252
Perinatal mortality/1000	41	43	1	34
Maternal deaths	14	6	0	6
Maternal mortality/100000	220	104	0	81
Caesarean sections	2412	2574	0	2574
Caesarean sections (%)	38%	45%	0	35%

## Discussion

These data are presented for illustrative purposes; we have not performed any statistical comparisons as these would not be meaningful, given the potential for bias in observational data. The high mortality and caesarean section rates reflect the fact that Frere is a high risk referral unit. In the South African public health system, the distinct separation of primary from secondary/tertiary services is usually geographic. There is an understandable reluctance of health authorities to have primary and secondary/tertiary services on the same site because of the risk of an inappropriate shift of low-risk cases to the higher level of care, with increased cost. However, as set out in this paper, care during labour is and must be an exception to this principle. We have shown that having the primary care services within the hospital can, in fact, result in a reduction in the number of births in the secondary/tertiary setting. Although the interpretation of the data from this audit is limited, it is possible that the OMBU contributed to the observed reduction in perinatal and maternal mortality rates. Other factors could also have contributed, such as a change in the patient profile, improved medical staffing, improvements in treatment for women with HIV due to the progressive government policy, and other unknown factors. What has become apparent to OU staff at Frere Hospital is that the quality of care in the OU has improved due to the reduced number of low-risk women giving birth. Furthermore, there appear to be many other advantages, both for staff and for the women using this service. We plan to conduct further research to evaluate these advantages and refine the model to further enhance the normal philosophy of childbirth. To evaluate whether the observed reductions in maternal and perinatal deaths represent real risk reductions, a cluster randomised trial would be necessary.

### The benefits of the OMBU model include:

- ▪ Low-risk pregnant women who present at the hospital in labour can be triaged directly to the OMBU, rather than being sent away to an off-site community health center or receiving inappropriate secondary level care in the hospital OU.
- ▪ Increased medical attention for the high-risk women in the less crowded hospital obstetric unit (OU).
- ▪ Greater capacity for more low-risk women to give birth on the hospital premises, thereby increasing their access to emergency obstetric and neonatal services in the event of complications.
- ▪ Timely management of complications arising in the OMBU by immediate transfer to the hospital service, or by consultation with an onsite OU doctor.
- ▪ Highly motivated staff in the OMBU due to the thriving nature of the unit, with large numbers of births and ready backup from the hospital OU.
- ▪ Increased quality of care for low-risk pregnancies due to the ability of motivated staff in the OMBU to focus on comfort and quality of midwifery care.
- ▪ Improved birth experience for women, leading to an improved reputation of the institution, which will encourage more women to make timely use of primary care services.

Midwife-led care has been shown to be more cost-effective than standard maternity care in two randomized controlled trials conducted in Australia and Norway [7,8]. Although we did not perform an economic evaluation, the OMBU would appear to be more cost-effective than the standard primary care model. This is because most primary care community health centres need a 24-hour staff complement, even if only one or two births take place each day. The OMBU model lends itself to greater cost-effectiveness due to economies of scale, with large number of women using the facility. Models of birth care whereby some low-risk women in large hospitals are allocated to a midwife birth, sometimes in a separate section of the OU, do not achieve the same levels of cost-effectiveness, as these low-risk women still incur the expense of an admission to a hospital. In addition, with this strategy the principle of the primary care model is undermined.

The fact that women try to avoid certain services, as described in the study from Tanzania referred to above [2] highlights the issue of women’s fear, the need for attention to quality of care in all services, and for further research in this field.

Finally, referrals from primary to secondary care are currently made according to conditions listed in national guidelines. If the primary care OMBU model is used, because of the close proximity to the doctor-led OU, there is significant scope to modify these guidelines for maternity care in South Africa, in order to improve the provision and experience of care.

## Conclusions

By incorporating many of the features of normalised midwife-led care with close proximity to doctor-led emergency care if required, the OMBU model has great potential to improve the quality of care provided in primary care settings in South Africa. There are many advantages for pregnant women, hospital staff and primary care staff with this model, which may also prove more cost-effective than the standard primary care model currently used in South Africa. A unique aspect of the OMBU is that it is staffed, administered and funded by the primary care services. We suggest that primary care OMBUs could be considered for any secondary/tertiary care hospitals in South Africa and possibly other countries with similar primary care health system models.

## Abbreviation

OMBU: Onsite midwife-led birth unit
OU: Obstetric unit
AMU: Alongside maternity unit
FMU: Free-standing maternity unit
